# Sputtering-driven formation of interstitial oxygen for intrinsic NIR detection in IGZO phototransistor

**DOI:** 10.1038/s41598-026-40769-z

**Published:** 2026-02-25

**Authors:** Jinsik Choe, Hyeonmin Bong, Huiyeong Lee, Dong-Hun Yeo, Sahn Nahm, In Soo Kim, Mann-Ho Cho, Kwangsik Jeong, Sungjin Park

**Affiliations:** 1 Semiconductor Solution Center, Icheon Branch, Korea Institute of Ceramic Engineering and Technology (KICET), Icheon, 17303 Republic of Korea; 2 Department of Materials Science and Engineering, Korea University, Seoul, 02841 Republic of Korea; 3 Department of Physics, Yonsei University, Seoul, Republic of Korea; 4 Center for Nanoscale Materials, Argonne National Laboratory, Lemont, IL 60439 USA; 5 Department of System Semiconductor Engineering, Yonsei University, Seoul, 03722 Republic of Korea; 6 Division of AI Semiconductor, Yonsei University, Wonju, 26493 Republic of Korea

**Keywords:** IGZO, phototransistor, NIR, On/off sputtering, Oxygen vacancy, interstitial oxygen, Sub-gap, Materials science, Nanoscience and technology, Optics and photonics, Physics

## Abstract

Amorphous indium gallium zinc oxide (a-IGZO) is a promising wide-bandgap semiconductor for large-area optoelectronics; however, its intrinsic insensitivity to sub-bandgap photons typically necessitates extrinsic dopants or heterostructures for near-infrared (NIR) photodetection. Here, we report a heterostructure-free and dopant-free broadband phototransistor that achieves intrinsic NIR sensitivity through geometry-driven defect engineering during sputter deposition. Amorphous IGZO thin films with a nominal In: Ga: Zn atomic ratio of ≈ 1:2:1 were deposited using on-axis (vertical) and off-axis (horizontal) sputtering configurations. While on-axis IGZO only exhibited visible-light photosensitivity, off-axis IGZO displayed a pronounced NIR response, enabled by the formation of interstitial oxygen (O_i_) shallow states. X-ray photoelectron spectroscopy (XPS) and composition-matched density functional theory (DFT) calculations confirmed that these O_i_-induced defect states lie 0.1–0.5 eV above the valence band maximum (VBM), effectively narrowing the optical bandgap and enabling sub-gap absorption and photogating under 850 nm illumination. The optimized a-IGZO phototransistor achieves a responsivity of 42.5 A W^-1^, an external quantum efficiency of 6.2 × 10^3^%, and a specific detectivity of 8.3 × 10^11^ Jones, all without plasmonic, hybrid, or quantum-dot sensitizers. Moreover, the off-axis process exhibits < 10% device-to-device variation across 5 samples, confirming its robustness and compatibility with large-area fabrication. To validate its practical utility, the off-axis IGZO device was further employed to quantify the sugar content (Brix) of coffee samples under NIR illumination, showing a clear correlation between photocurrent and concentration. This work demonstrates a simple, scalable, and CMOS-compatible approach to extending the spectral response of oxide semiconductors, opening new opportunities for cost-effective broadband photodetectors and integrated photonic systems.

**Supplementary Information:**

The online version contains supplementary material available at 10.1038/s41598-026-40769-z.

## Introduction

Phototransistors with sensitivity extending into the near-infrared (NIR) region have attracted significant attention due to their wide applicability in optical communication, biomedical diagnostics, and non-invasive food quality monitoring^1–6^. In particular, the food and beverage industry has an increasing demand for rapid, non-destructive sensing platforms to quantify soluble solids, such as sugar content (Brix), in complex liquid media. NIR light is uniquely suited for these applications as it can penetrate deep into dark-colored or turbid samples such as brewed coffee, where conventional visible light is often obstructed by high absorption and scattering^6^. Amorphous indium gallium zinc oxide (a-IGZO) has emerged as a premier semiconductor for next-generation optoelectronics due to its high electron mobility, low off-state current, and uniform electronic properties^7–17^. Despite these advantages, the practical use of IGZO for NIR photodetection is fundamentally limited by its wide optical bandgap, typically ranging between 3.0 and 4.0 eV, which results in negligible absorption for wavelengths beyond 500 nm. Consequently, pristine IGZO is inherently unsuitable for broadband photodetection without further material modification to introduce sub-gap states^18–23^. To overcome this limitation, various approaches have been explored, including the integration of IGZO with low-bandgap sensitizers such as quantum dots, organic semiconductors, or two-dimensional layered materials^24–27^. Furthermore, extrinsic doping with elements such as Ag, Cu, or Nb has been employed to create sub-gap energy levels^28–30^. While effective, these hybrid architectures significantly increase fabrication complexity, production costs, and often introduce interface stability issues or degradation of electrical performance. Particularly for large-scale manufacturing, increasing process complexity directly impacts yield and scalability, necessitating a more simplified and cost-effective solution.

To address these challenges, we introduce an alternative, heterostructure-free and dopant-free strategy that activates NIR response through geometry-driven defect engineering during sputter deposition. By simply modulating the sputtering orientation from a conventional on-axis to an off-axis configuration, we selectively stabilize interstitial oxygen (O_i_) shallow states within the a-IGZO matrix. This approach is exceptionally advantageous as it achieves broadband sensitivity using only native defect control, ensuring a simple, scalable, and CMOS-compatible process that preserves material purity without the need for additional materials or complex vacuum-breaking steps. In this study, we demonstrate that off-axis IGZO films intrinsically generate shallow localized states approximately 0.1–0.5 eV above the valence band maximum (VBM). These O_i_-induced states act as stable sub-gap absorption centers, enabling efficient trap-assisted photogating under 850 nm illumination. To highlight its practical utility, the optimized NIR-sensitive phototransistor was successfully employed to differentiate sugar concentrations (Brix) in brewed coffee. The device exhibited a clear, quantifiable correlation between photocurrent and concentration, demonstrating significantly enhanced accuracy compared to commercial refractometers in high-Brix ranges. These results prove that sputtering geometry alone can serve as a powerful tool for tuning the optoelectronic range of oxide semiconductors, paving the way for cost-effective smart sensing platforms.

## Results and discussion

**Fig. 1:**
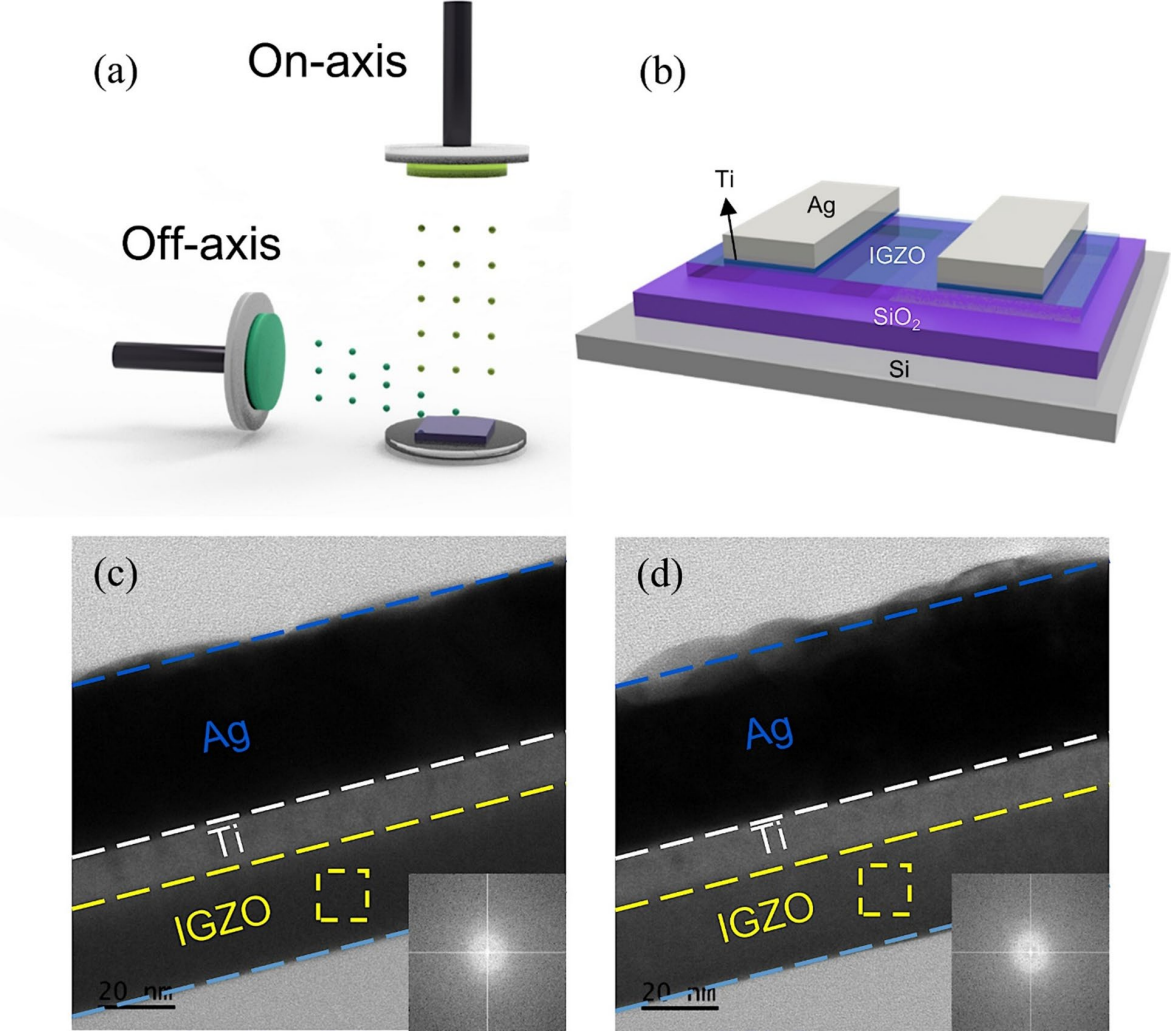
Concept of on/off-axis sputter & device morphology. (**a**) On- and off-axis sputter. (**b**) IGZO phototransistors deposited by on- and off-axis sputter. TEM images and SAED patterns (**c**) On-, (**d**) Off-axis IGZO.

A schematic of the deposition process for IGZO thin films using the on/off-axis sputtering method is shown in Fig. 1a. In the on-axis sputtering method, the target is positioned above the substrate during the deposition. In the off-axis sputtering method, the target is positioned to the side of the substrate during the deposition. Variations in sputtering methods can lead to differences in the energy imparted to the thin film during the deposition process, influencing not only the crystallinity of the film but also the formation of defects within the film^31^. Here, all plasma parameters were fixed, and only the target orientation was varied, enabling us to attribute the NIR response to geometry driven oxygen-defect modulation. The device structure of IGZO is shown in Fig. 1b. A highly doped silicon wafer serves as both the substrate and the back gate, with Ti/Ag metals used for the source and drain. Figure 1c and d show the TEM images of devices fabricated with on- and off-axis IGZO, respectively. The IGZO, Ti, and Ag layers are each well-formed without any inter-diffusion between the layers (Supporting Figure S1). As can be seen in the inset figure, the Selected Area Electron Diffraction (SAED) pattern confirms that the IGZO thin film exhibits an amorphous structure.

**Fig. 2:**
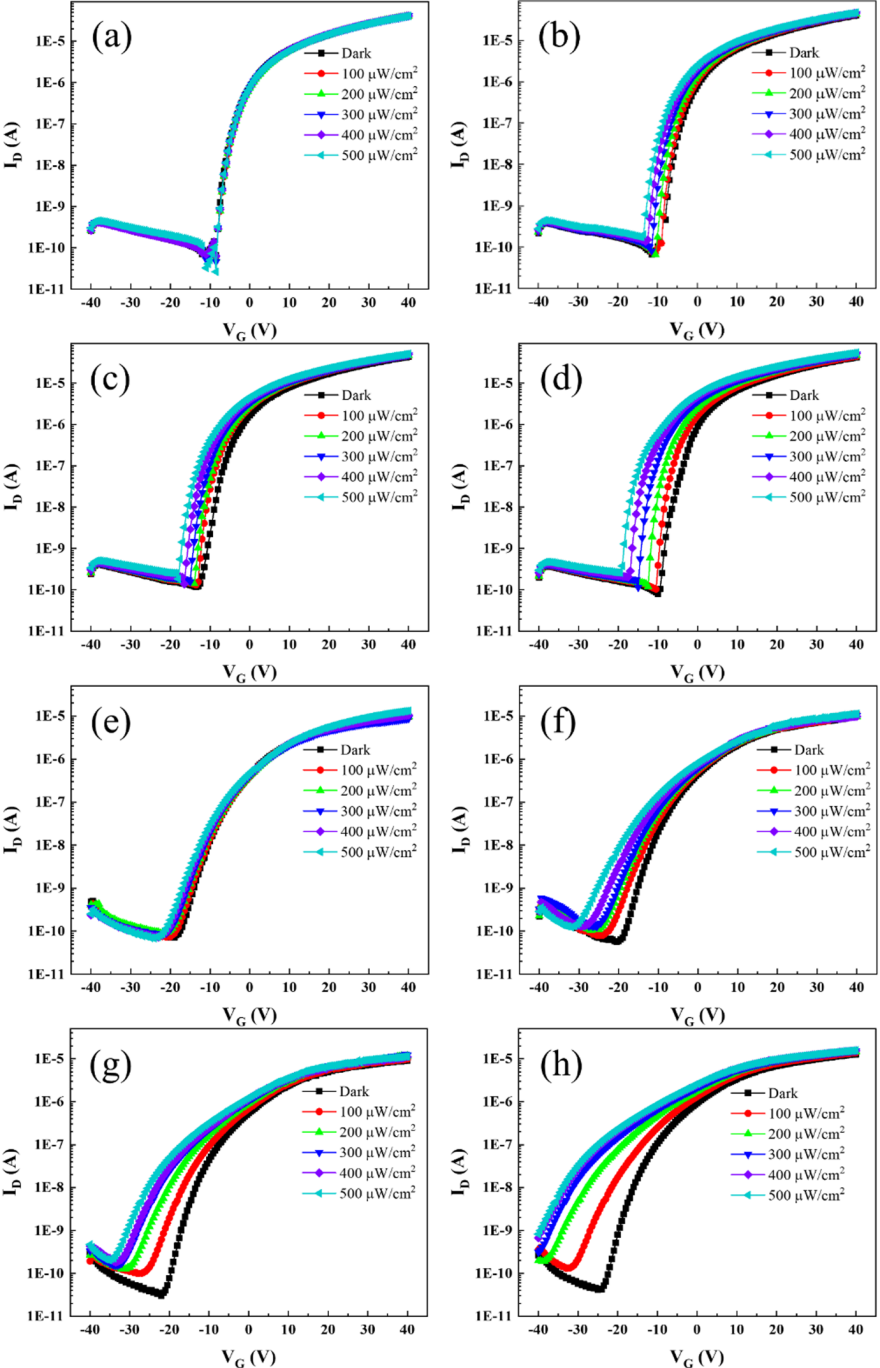
Photoresponse of the IGZO phototransistor under incident light of 850 (NIR), 625 (RED), 530 (GREEN), 470 (BLUE) nm. On-axis IGZO split according to power density. (**a**) NIR, (**b**) RED, (**c**) GREEN, (**d**) BLUE. Off-axis IGZO split according to power density. (**e**) NIR, (**f**) RED, (**g**) GREEN, (**h**) BLUE.

To confirm the electrical property of on-axis/off-axis IGZO device, the transfer curve was measured from − 40 V to 40 V of V_g_ with V_d_=5V. The on-axis IGZO device exhibits a V_th_ of -8.5 V, a *µ*_sat_ of 5.4 cm²/Vs, a subthreshold swing of 0.78 V/dec, and an on/off ratio of 1.16 × 10^5^ (as shown in Fig. 2a and Figure S2). In contrast, the off-axis IGZO device shows a V_th_ of -20 V, a *µ*_sat_ of 0.6 cm^2^/Vs, a subthreshold swing of 2.97 V/dec, and an on/off ratio of 1.85 × 10^5^ (as shown in Fig. 2e, Figure S2, and Table S1). To verify the reproducibility of the electrical and optical characteristics, multiple on- and off-axis IGZO phototransistors were fabricated and characterized under identical sputtering and annealing conditions. As illustrated in Figure S3, both device types exhibited nearly identical electrical transfer characteristics (V_g_ – I_d_) in the dark, showing less than 10% variation in threshold voltage among five independently fabricated samples. This confirms the high reproducibility of the electrical performance for both on- and off-axis IGZO devices under identical deposition and annealing conditions. To evaluate the photoresponse property of on-/off-axis IGZO devices, the transfer characteristics were measured under laser irradiation of 850(NIR), 625(red), 530(green), 470(blue) nm and within a power range from 100 to 500 µW cm^-2^. The transfer characteristics of the on-axis IGZO device under varying wavelengths are shown in Fig. 2a, b, c, and d, respectively. The transfer characteristics of the off-axis IGZO device under the same light conditions are displayed in Fig. 2e, f, g, and h. Detailed magnified views of the threshold voltage (V_th_) shifts are provided in Figure S4 of the Supporting Information. Both on- and off-axis IGZO devices exhibit photoresponse characteristics, showing V_th_ shifts under blue, green, and red laser irradiation. Under red, green, and blue laser irradiation, the on-axis IGZO device exhibited V_th_ shift of -4.5, -5.5, and − 9.5 V at 500 µW cm^-2^, respectively, while the off-axis IGZO device exhibited V_th_ shift of -12, -13, and more than − 16 V at 500 µW cm^-2^, respectively. The V_th_ shift in the off-axis IGZO device is larger than that in the on-axis IGZO device. In particular, the two devices behave markedly differently under NIR laser irradiation. The on-axis IGZO device shows virtually no detectable photoresponse, whereas the off-axis device produces a distinct and reproducible signal. Furthermore, as the laser power increased, the degree of photoresponse also increased (ΔV_th_ shift = -5 V). Furthermore, the reversibility of the photo induced changes in the transfer characteristics of the off-axis IGZO phototransistor (Figure S5) was carefully verified by monitoring the baseline dark current after each illumination sweep. For the 850 nm (NIR) illumination, the dark current exhibited negligible change, returning almost perfectly to its initial state. While minor fluctuations were observed following exposure to visible wavelengths (Red, Green, and Blue), these variations remained well within the experimental margin of error and did not indicate any irreversible degradation of the off-axis device. As shown in Figure S6, the transient photoresponses were observed for each laser wavelength and power. A distinct photoresponse was recorded throughout the laser on/off cycles. Furthermore, operational stability was confirmed by repeated on/off cycling (Figure S6). The devices exhibited consistent and reproducible photoresponses without significant degradation over multiple cycles. To further quantify the response speed of the a-IGZO phototransistors, the rise time (*τ*_*r*_) and fall time (*τ*_*f*_) were extracted from the transient curves (Figure S6), defined as the time interval required for the photocurrent to transition between 10% and 90% of its peak value. For the off-axis device under 850 nm illumination, the extracted *τ*_*r*_ and *τ*_*f*_ were approximately 4.1 s and 10 s, respectively. The notably longer fall time compared to the rise time is attributed to the persistent photoconductivity (PPC) effect, which originates from the slow recombination of photogenerated carriers trapped in O_i_-induced shallow states. This PPC behavior is a characteristic phenomenon typically associated with trap-mediated gain mechanisms, such as photogating or photodoping. To verify the presence of these mechanisms, we analyzed the dependence of photocurrent on the incident optical power density under NIR illumination. As shown in Figure S7, the photocurrent follows a sublinear power-law relationship (I_ph_ ∝ P_in_^α^) with an extracted exponent of α = 0.84. This sublinear scaling (α < 1) provides strong evidence for a photogating or photodoping-dominated response, where the photoexcited carriers are captured by O_i_ defect states to modulate the effective gate potential^32–35^. The deviation of α from unity further confirms that carrier trapping and release processes within the shallow states govern the photoresponse, rather than a direct band-to-band excitation process. This observation highlights a characteristic trade-off where the oxygen-defect-driven trap states significantly enhance NIR responsivity at the cost of moderate response speed. In addition to the mechanism analysis, the long-term reliability of the NIR-sensitive phototransistor was evaluated through 10 days aging study. As shown in Supporting Figure S8, the off-axis IGZO device was re-characterized after being stored in an ambient atmosphere for 10 days. The results demonstrated that the transfer characteristics and the NIR-induced photoresponse remained remarkably stable, with no discernible degradation or baseline drift observed over the 10-day period. Specifically, a significant negative Vth shift remained clearly visible under NIR illumination showed consistent switching cycles with well-preserved photocurrent levels. These results confirm that the geometry-driven NIR sensitivity is a structurally stable property, ensuring the operational reliability of the proposed sensing platform. Notably, the off-axis IGZO device maintained a distinct and stable photoresponse under NIR laser irradiation. Generally, IGZO possesses a bandgap that exceeds 3 eV, limiting its intrinsic photoresponse to the visible spectrum and rendering it inactive in the NIR range^13^. As a result, numerous studies have introduced complex approaches, such as heterojunction formation and micro-patterning, to extend the photoresponse of IGZO devices into the broadband (Vis.–NIR) spectrum^14–20^. In contrast, we demonstrate an effective NIR photoresponse in IGZO by employing a straightforward off-axis sputtering deposition technique. This approach enables the realization of NIR sensitivity without the need for additional materials or intricate patterning processes. Consequently, we systematically investigate the differences in the properties of IGZO thin films prepared via off-axis sputtering compared to those deposited through conventional on-axis sputtering, revealing the significant impact of deposition geometry on photoresponse characteristics.

**Fig. 3:**
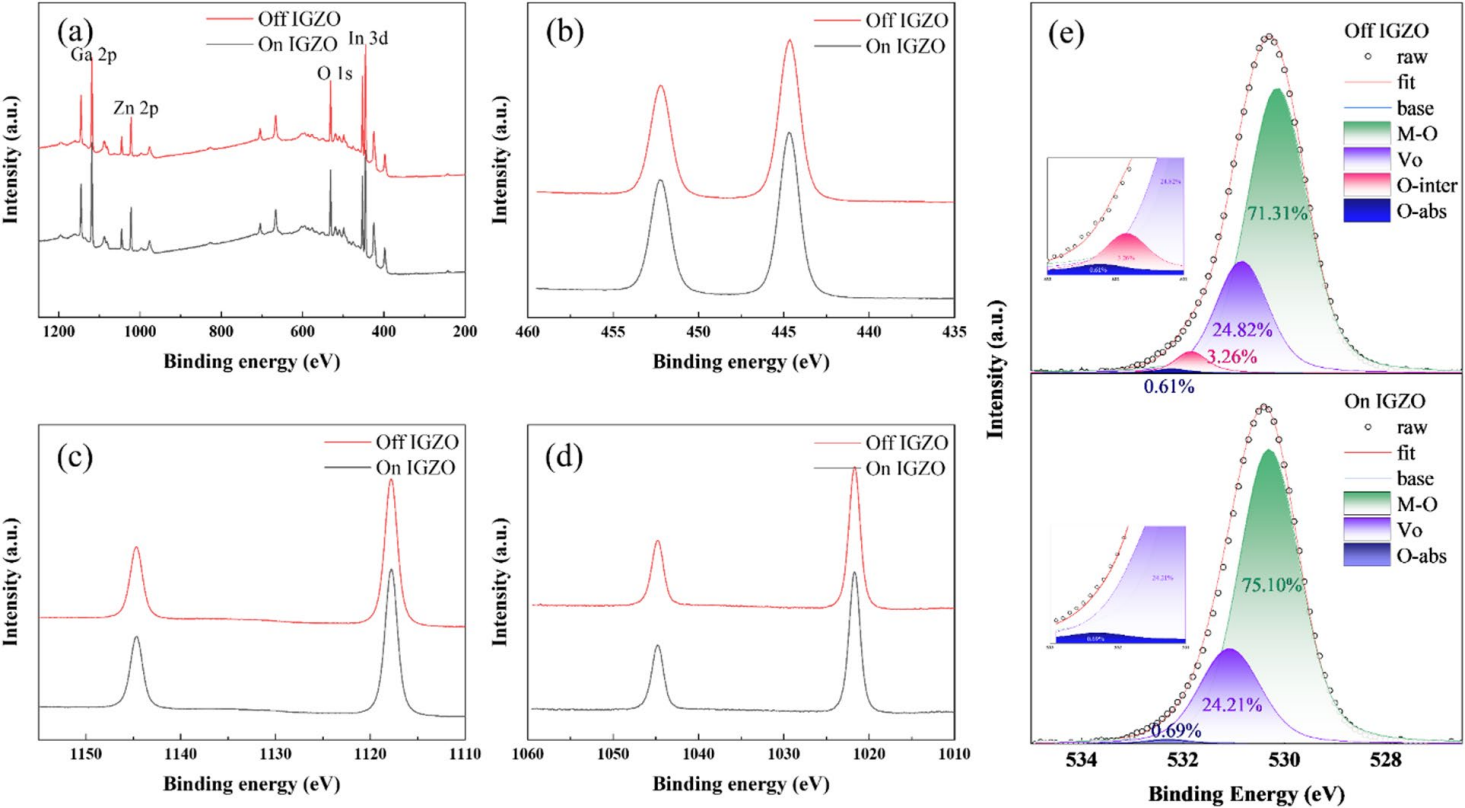
Chemical composition of On- and Off-axis IGZO using XPS. (a) Low-resolution survey spectra confirming the presence of In, Ga, Zn, and O elements. High-resolution deconvoluted spectra of core level (**b**) In 3d, (**c**) Ga 2p, (**d**) Zn 2p, and (**e**) O 1s for both On and Off IGZO.

XPS measurements were conducted to examine the differences in composition and chemical bonding between on- and off-axis IGZO. The presence of In, Ga, Zn, and O in both on- and off-axis IGZO was confirmed from the low-resolution survey spectra (Fig. 3a), consistent with the EDS mapping results (Figure S1). The composition of on- and off-axis IGZO shows slight differences at 13.33:24.24:8.9:53.53 and 11.66:24.47:9.27:54.6, respectively. However, considering the resolution of XPS, these compositional differences are not statistically significant. The chemical properties of individual elements were analyzed through deconvolution of the high-resolution spectra (Fig. 3b–e, Figure S9). An internal calibration using C 1s at 285 eV was conducted, and Ar sputtering was employed to remove native surface oxidation. A comparison of the In 3d, Ga 2p, Zn 2p, and O 1s core levels of on- and off-IGZO showed no significant differences in the In 3d, Ga 2p, and Zn 2p spectra. In the In 3d spectrum, the In 3d5/2 and In 3d3/2 peaks of on- and off-IGZO appeared at 444.7 eV and 452.21 eV, respectively. In the Ga 2p spectrum, the Ga 2p3/2 and Ga 2p1/2 peaks of on- and off-IGZO were observed at 1144.7 eV and 1117.8 eV, respectively. In the Zn 2p spectrum, the Zn 2p3/2 and Zn 2p1/2 peaks appeared at 1044.8 eV and 1021.7 eV for both on- and off-IGZO. Additionally, there were no significant differences in the deconvoluted spectra (in Figure S9). In the O 1s spectrum, there was a difference of approximately 0.1 eV in the peak positions between on- and off-IGZO. Moreover, the deconvoluted spectrum revealed three components in on-IGZO and four components in off-IGZO. Typically, in the O 1s spectrum, 530 eV corresponds to metal-oxygen (MO) bonding, 531 eV to oxygen vacancies, 531.5 eV to interstitial oxygen, and 532 eV to surface adsorption^36–38^. In on-axis IGZO, the deconvolution results showed that MO bonding accounted for 75.10% of the O 1s peak, oxygen vacancies for 24.21%, and surface adsorption for 0.69%. Notably, no significant interstitial oxygen was observed in on-axis IGZO. In contrast, off-axis IGZO exhibited different proportions. MO bonding accounted for 71.31%, oxygen vacancies for 24.82%, interstitial oxygen for 3.26%, and surface adsorption for 0.61%. Although the off-axis IGZO exhibited a slightly higher oxygen vacancy content (24.82%) than the on-axis IGZO (24.21%), this difference is insufficient to account for the significant enhancement in photoresponse. Therefore, oxygen vacancies alone cannot explain the observed NIR sensitivity, implying the presence of additional defect states that play a more dominant role in facilitating sub-gap absorption. In particular, the localized presence of interstitial oxygen in the off-axis IGZO contributes to forming a more complex and heterogeneous oxygen environment within the film. The presence of interstitial oxygen is likely to introduce sub-gap states into the band structure, creating shallow trap levels that can be activated by low-energy photons such as those in the NIR range. In addition, XPS quantification showed that, although the sputtering target had a nominal In: Ga: Zn composition of 1:1:1, the deposited IGZO films exhibited a cation ratio of approximately 1:2:1 for both on- and off-axis configurations. This measured stoichiometry was therefore used in our DFT calculations to ensure direct comparability between the calculated defect energetics and the experimental chemical states.

**Fig. 4:**
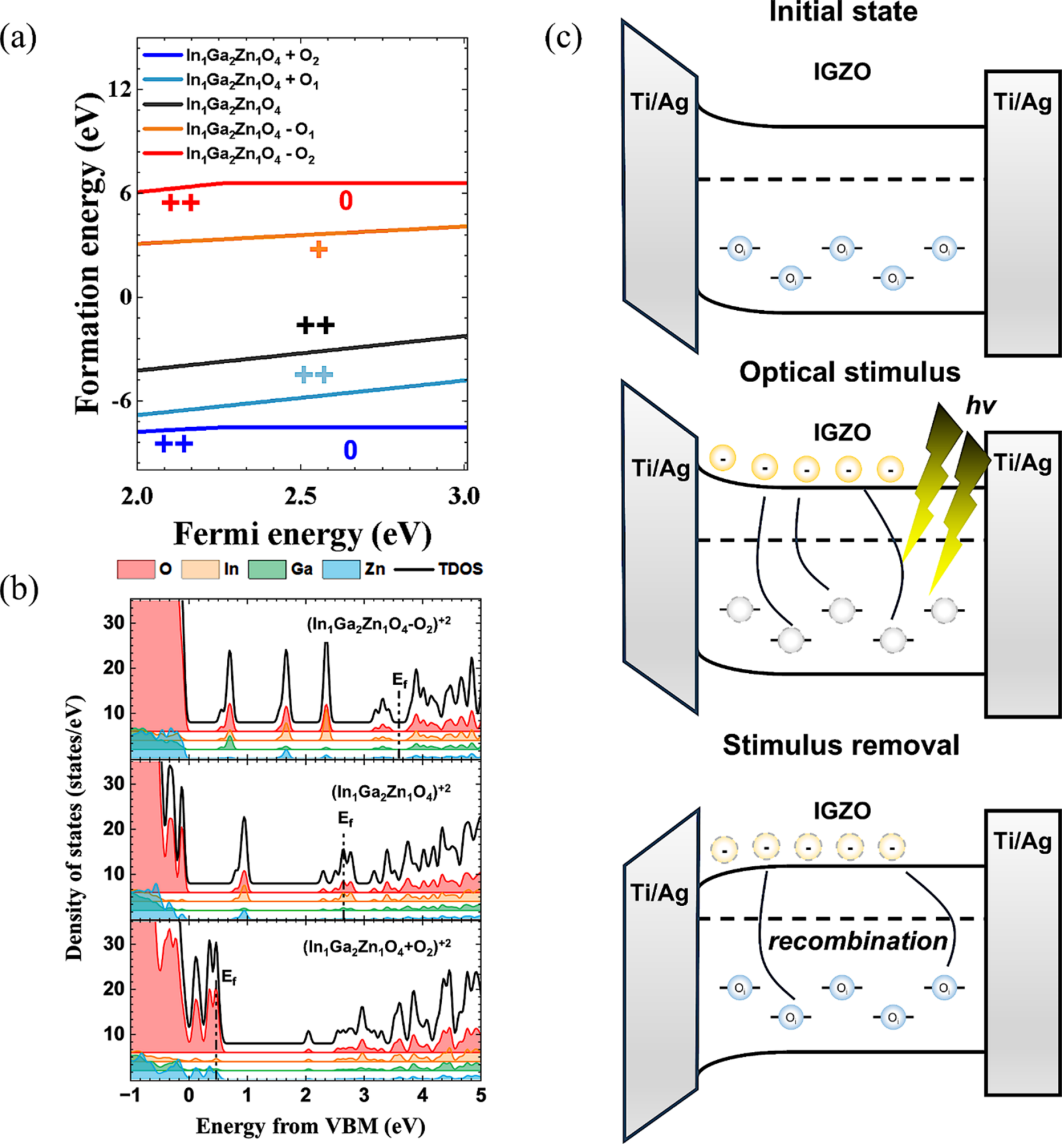
(**a**) Formation energy for IGZO (**b**) density of states for IGZO. (**c**) Schematic illustration of band diagrams to explain the NIR-response process in off-axis IGZO device.

Figure 4a presents the formation energies of IGZO with different oxygen configurations, and the details of the DFT calculations based on this model are discussed in the Experimental Methods sections^39–42^. To accurately reflect the experimentally determined stoichiometry (In: Ga: Zn = 1:2:1), an amorphous In_1_Ga_2_Zn_1_O_4_ model was used for the calculations. This compositional agreement between experiment and model allows direct comparison between the calculated defect energetics and the measured chemical states. The black line corresponds to the ideal IGZO composition (In_1_Ga_2_Zn_1_O_4_), while the yellow and red lines represent oxygen-deficient configurations with one (In_1_Ga_2_Zn_1_O_4_-O_1_) and two (In_1_Ga_2_Zn_1_O_4_-O_2_) oxygen vacancies, respectively. The green and blue lines correspond to oxygen-rich conditions with one (In_1_Ga_2_Zn_1_O_4_+O_1_) and two (In_1_Ga_2_Zn_1_O_4_+O_2_) excess oxygen atoms, respectively. Generally, in IGZO systems with an In: Ga: Zn ratio of 1:1:1, a small number of oxygen vacancies are energetically stable and act as donors by carrying positive charges. However, in the 1:2:1 system, oxygen vacancies are energetically less favorable compared to the ideal state. Instead, oxygen-rich configurations become more thermodynamically stable. In particular, the In_1_Ga_2_Zn_1_O_4_+O_2_ state exhibits the lowest formation energy, indicating the highest stability. This phenomenon can be explained by the chemical bonding characteristics of the cations. Among the constituent cations, Gallium (Ga) exhibits the highest bonding energy with oxygen (~ 2.0 eV), far exceeding the bond strengths of In-O or Zn-O^43^. Consequently, the Ga-rich stoichiometry (In: Ga: Zn = 1:2:1) employed in this study effectively serves as an ‘oxygen getter’ during the sputtering process, promoting a localized oxygen-rich environment by capturing excess oxygen from the plasma. As evidenced by our DFT calculations (Fig. 4a), this oxygen-rich stoichiometry (In_1_Ga_2_Zn_1_O_4_+O_2_) is thermodynamically preferred, exhibiting the lowest formation energy among various configurations in the Ga-rich system. Within this stable oxygen-rich matrix, the excess oxygen is naturally accommodated as interstitial oxygen (O_i_), creating the sub-gap states necessary for NIR detection. The realization of this state is critically enabled by the off-axis sputtering geometry. While the high-energy bombardment characteristic of on-axis sputtering leads to the ‘resputtering’ of oxygen species and disrupts the stabilization process, the off-axis configuration provides a kinetically gentle environment that minimizes such ionic damage^44^. This allows the thermodynamically favored oxygen-rich state and its constituent O_i_ defects to be stably preserved within the robust amorphous network. Figure 4b and Figure S10 show the density of states (DOS) calculations for various charge states. According to the DOS analysis, the Fermi level in the oxygen-deficient sample (In_1_Ga_2_Zn_1_O_4_-O_2_) and the ideal IGZO (In_1_Ga_2_Zn_1_O_4_) are located at 3.59 eV and 2.67 eV, respectively. In contrast, the Fermi level in the oxygen-rich configuration (In_1_Ga_2_Zn_1_O_4_+O_2_) appears near the valence band maximum (VBM), specifically at 0.47 eV above the VBM. The trap states are located at 0.55 eV, 0.69 eV and 1.66 eV above the valence band for (In_1_Ga_2_Zn_1_O_4_-O_2_)^+2^, and at 0.80 eV, 0.94 eV and 2.29 eV for (In_1_Ga_2_Zn_1_O_4_)^+2^. In both cases, there is a deep defect level. In contrast, the oxygen-rich configuration (In_1_Ga_2_Zn_1_O_4_+O_2_) intrinsically generates a series of shallow localized states at approximately 0.12, 0.35, and 0.47 eV above the VBM, as shown in Fig. 4b and Figure S10. These states arise from the metastable incorporation of interstitial oxygen within the amorphous IGZO network, where the excess oxygen atom distorts nearby Ga–O and In–O bonds. Owing to this incorporation, interstitial oxygen naturally forms partially occupied trap levels even without external bias. Upon NIR illumination, electrons confined in these O_i_-induced shallow states undergo trap-assisted transitions. Rather than a direct transition to the CBM, these carriers are redistributed among the sub-gap states, effectively facilitating an intrinsic pathway for sub-gap absorption. Therefore, the NIR photoresponse observed in off-axis IGZO originates from these metastable interstitial oxygen sites, rather than from field-induced or vacancy-related effects. As a result, IGZO deposited under ideal conditions corresponding to on-axis sputtering cannot absorb IR light efficiently and thus shows negligible changes in electrical signal under IR illumination. Therefore, the off-axis IGZO, which exhibits oxygen-rich characteristics consistent with the DFT model, is likely to contain interstitial oxygen that introduces shallow donor-like states. These states mediate trap-assisted absorption of low-energy photons, resulting in the observed NIR photoresponse. This interpretation is consistent with the presence of interstitial oxygen observed in Fig. 3e and the NIR-responsive behavior shown in Fig. 2e. Moreover, the formation energy curve for In_1_Ga_2_Zn_1_O_4_+O_2_ (blue line in Fig. 4a) exhibits an inflection point. This inflection is indicative of changes in the material’s defect states, which may act as charge traps. Under light irradiation, off-axis IGZO displays subtle variations in the slope of its transfer curve (Supporting Figure S4), further supporting the presence of electrically active defect states. Based on electrical characterization, chemical bonding analysis, and DFT simulations, we conclude that interstitial oxygen is present in off-axis IGZO. As illustrated in Fig. 4c, O_i_-induced shallow states are located 0.1–0.5 eV above the VBM and are partially occupied even without external bias. Under NIR illumination, carriers can be redistributed among these shallow states and higher-lying intrinsic sub-gap tail states, effectively reducing the energy required for electronic transitions as depicted in recent sub-gap transition models. This leads to a photogating-induced modulation of the channel conductivity, a phenomenon that has been validated for NIR detection in specialized IGZO nanostructures^33^. This interpretation links the DFT-predicted shallow defect distribution with the experimentally observed broadband responsivity, highlighting that geometry-driven oxygen incorporation alone can intrinsically extend the spectral range of IGZO phototransistors. The optoelectronic performance of the optimized off-axis a-IGZO phototransistor was evaluated under 850 nm illumination. The responsivities (*R*) of the phototransistors were calculated using Eq. (1):

1 $$\:R=\frac{{I}_{ph}}{{P}_{in}}$$

where *I*_ph_ is the photocurrent (*I*_light_ - *I*_dark_) and P_in_ is the incident light power. To evaluate the specific detectivity (D^*^), we employed the following general relationship^45^:

2 $$\:{D}^{*}=\:\frac{{R\:\:A}^{1/2}}{{i}_{noise}}$$

where A is the effective photosensitive area, and *i*_noise_ is the noise current. In this study, assuming that the noise is dominated by the shot noise from the dark current (I_dark_), the noise current can be expressed as *i*_noise_ = (2qI_dark_)^1/2^, where q is the elementary electronic charge. Under this assumption, D* can be simplified as follows^1,45–47^:

3 $$\:{D}^{*}=\:\frac{{R\:\:A}^{1/2}}{{\left(2q{I}_{dark}\right)}^{1/2}}$$

While this shot-noise-limited model is widely used for performance comparison in oxide semiconductor photodetectors, it represents a theoretical limit. The device exhibited a responsivity of 42.5 A W^-1^, an external quantum efficiency (EQE) of 6.2 × 10^3^%, and a specific detectivity (D*) of 8.3 × 10^11^ Jones. These values are comparable to or surpass those of previously reported IGZO-based infrared photodetectors that rely on quantum-dot or plasmonic sensitizers (Table 1), confirming that geometry-driven defect formation alone can achieve high NIR sensitivity without extrinsic modifications.

**Table 1:** Figures of merit of IGZO material-based infrared photodetectors.

Material/structure	Wavelength (nm)	*R* (A/W)	EQE (%)	D^*^ (Jones)	References
PbS QDs/IGZO + Al_2_O_3_ infilling	880	1.65 × 10^2^	117	1.11 × 10^13^	^1^
a‑IGZO phototransistor	980	1.2 × 10^5^	1.52 × 10^7^	2.13 × 10^15^	^10^
IGZO/PbS QD hybrid	850	19.1	2.25 × 10^3^	1.53 × 10^13^	^25^
IGZO/PbS QD hybrid	1550	196.7	–	5.47 × 10^12^	^46^
PbS QD/ZnO hybrid phototransistor	1550	1.87 × 10^2^	–	2.81 × 10^12^	^47^
PbS QDs/IGZO hybrid	800–1050	> 2.0 × 10^4^	–	> 1 × 10^14^	^48^
Ge-CL/ IGZO‑TFT IR sensor	NIR–IR	4.1 × 10^2^	–	2.3 × 10^14^	^49^
IGZO	850	42.5	6.20 × 10^3^	8.34 × 10^11^	This work

**Fig. 5:**
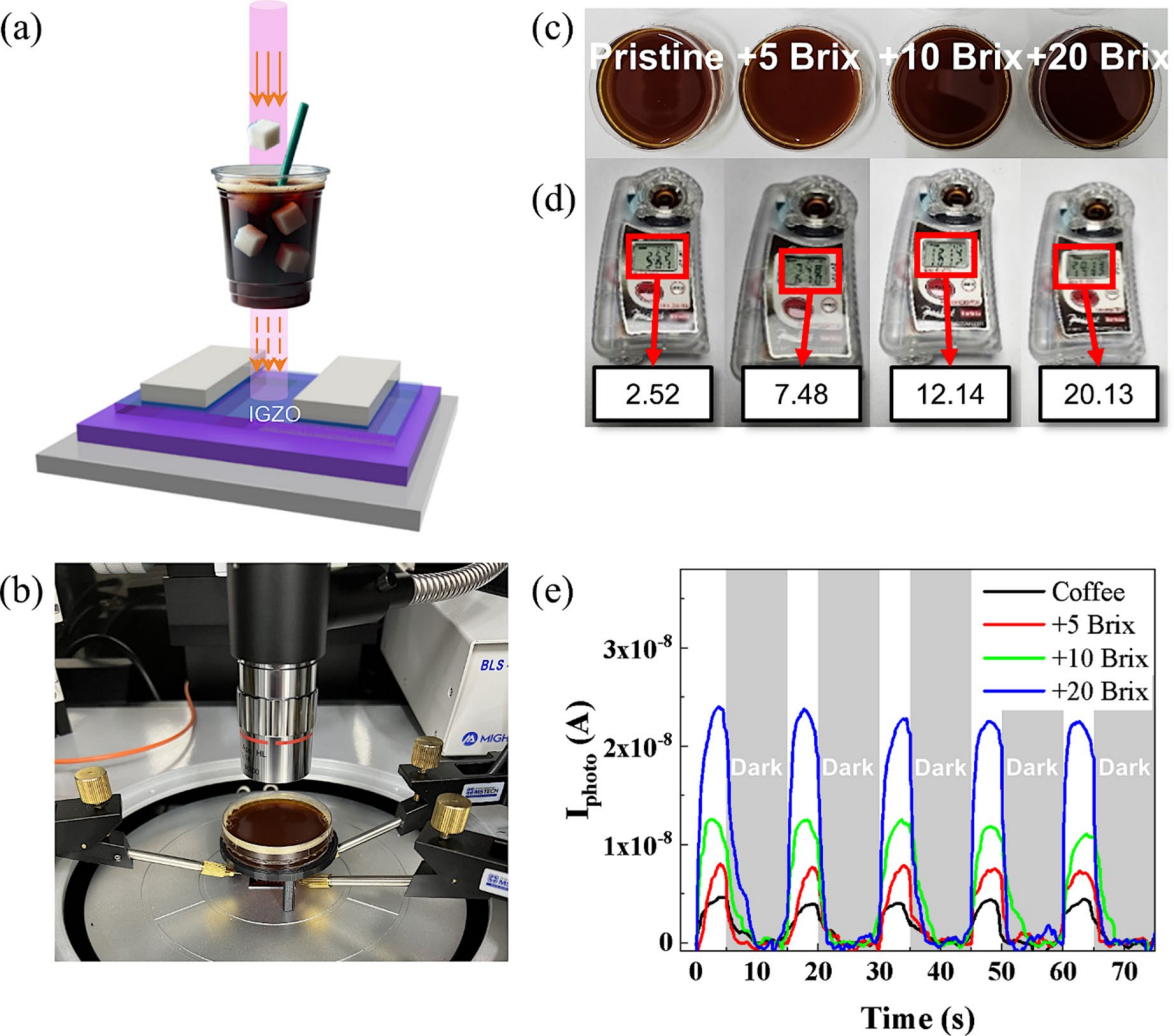
Experiment for application in measuring coffee Brix. (**a**) Concept of coffee Brix measurement. (**b**) Photograph of Brix measurement based on off-axis IGZO phototransistor. (**c**) Sugar-free coffee and coffee with added sugar of 5, 10, or 20 Brix. (**d**) Brix measurement in coffee using a commercial coffee refractometer. (**e**) Changes in transient photoresponse of off-axis IGZO phototransistor according to differences in Brix under incident light of 850 nm (NIR).

To evaluate the practical applicability of the off-axis IGZO device featuring near-infrared (NIR) photoresponse characteristics, variations in sugar content (Brix) of coffee samples were measured. Figures 5a, b present a schematic diagram and a photograph of the experimental setup for Brix measurement, respectively. As shown in Fig. 5c, four coffee samples were prepared. unsweetened Americano as a base, and three variants with added sugar corresponding to increased Brix levels of + 5, +10, and + 20. The precise Brix values of these samples were quantified using a commercial coffee refractometer (PAL-COFFEE, Atago Co., Ltd.) (Fig. 5d). The measured base Brix value for unsweetened Americano coffee was 2.52, while measured values for the + 5, +10, and + 20 Brix samples were 7.48, 12.14, and 20.13, respectively. If the unsweetened coffee is assumed to have a base Brix of 2.52, the expected Brix values for the + 5, +10, and + 20 samples would be 7.52, 12.52, and 22.52, respectively. While the + 5 Brix sample closely matched the expected value, the + 20 Brix sample exhibited a deviation of more than 10% from the expected concentration. To assess the performance of the on-/off-axis IGZO device developed herein, measurements were performed using the same set of coffee samples. Figure 5e shows the current change in coffee sugar content using the off-axis IGZO device under NIR illumination. Due to the dark color of the coffee samples, under green or blue illumination, the device did not produce any detectable photoresponse. Under red light, a photoresponse was detected. However, there is no clear correlation between the photoresponse and sugar content(Supporting Figure S11). Under NIR illumination, the on-axis IGZO device exhibited no detectable response, regardless of the coffee’s Brix level (Supporting Figure S6a). In contrast, the off-axis IGZO device displayed a clear and quantifiable photoresponse under NIR illumination, with the signal intensity varying distinctly according to the Brix level. The current value for the base coffee sample (2.52 Brix) is 3.95 × 10^− 9^ A. The current values for the + 5, +10, and + 20 Brix coffee samples were measured to be 8.02 × 10^− 9^, 1.26 × 10^− 8^, and 2.24 × 10^− 8^ A, respectively. The corresponding Brix values were then calculated using the relation Brix = 1.092 × 10^8^ × I – 1.79, yielding 6.97, 11.97, and 22.67 Brix, respectively. These calculated values correspond to measurement accuracies of 7.34%, 4.40%, and 0.67% relative to those obtained using the commercial refractometer. Notably, in the high-concentration (+ 20 Brix) coffee sample, the IGZO device demonstrated significantly enhanced measurement accuracy compared to the commercial refractometer. While the coffee Brix measurement serves as a successful proof of concept for liquid-phase sensing, the potential applications of this geometry-driven NIR-sensitive a-IGZO phototransistor extend far beyond food quality monitoring. Given the high uniformity and reproducibility (< 10% device-to-device variation), this technology is particularly well-suited for large-area sensing arrays and high-resolution imaging systems where consistent performance across a wide substrate is critical. Furthermore, the heterostructure-free and dopant-free nature of the process ensures seamless integration with existing CMOS-based electronic systems, as it bypasses the material purity and interface stability issues often encountered in hybrid architectures like those summarized in Table 1. Unlike previously reported IGZO-based NIR detectors that rely on external sensitizers such as PbS quantum dots or organic semiconductors, our device achieves comparable performance (*R* = 42.5 A W^-1^, D^*^ = 8.3 × 10^11^ Jones) through a single-step, intrinsically controlled sputtering process. This offers a decisive advantage in terms of cost-effectiveness, fabrication scalability, and long-term stability, establishing a versatile platform for broadband oxide optoelectronics.

## Conclusion

In conclusion, we have demonstrated that a simple change in sputtering geometry enables intrinsic near-infrared (NIR) photoresponse in amorphous IGZO phototransistors without the need for heterostructures, dopants, or external sensitizers. The off-axis sputtering configuration generates a higher concentration of interstitial oxygen (O_i_), as confirmed by X-ray photoelectron spectroscopy (XPS), and density functional theory (DFT) calculations reveal that these O_i_ species introduce shallow defect states located 0.1–0.5 eV above the valence band maximum (VBM). These defect states act as sub-gap absorption centers, enabling trap-assisted photogating under 850 nm irradiation and effectively extending the spectral response of IGZO into the NIR region. Unlike oxygen vacancies, which do not correlate directly with NIR absorption, interstitial oxygen plays a dominant role in modulating the electronic structure and enhancing photoresponse. The resulting a-IGZO phototransistor achieves a responsivity of 42.5 A W⁻¹, an external quantum efficiency of 6.2 × 10³ %, and a detectivity of 8.3 × 10¹¹ Jones, with < 10% device-to-device variation across 5 samples. To further demonstrate the device’s practical applicability, the NIR-sensitive off-axis IGZO phototransistor was successfully employed to quantify the sugar content (Brix) of coffee samples, exhibiting a clear and linear correlation between photocurrent and concentration. These results demonstrate the scalability and reproducibility of the off-axis process and highlight defect engineering via geometry-controlled sputtering as a versatile and CMOS-compatible route to broadband oxide optoelectronics. This approach offers a new pathway for developing cost-effective photodetectors and smart sensing platforms capable of operating across the visible and NIR spectral regions.

## Experimental section

### Device fabrication

On- and off-axis IGZO layers were individually deposited onto a thermally grown SiO_2_ (300 nm)/p-type Si substrate (2 × 2 cm²) using vertically and horizontally positioned targets. Both amorphous IGZO films were prepared under identical sputtering conditions (RF power = 100 W, pressure = 5 mTorr, Ar/O_2_ = 45/2.5 sccm), ensuring stable and reproducible fabrication. The composition of the IGZO targets (3 inch diameter) utilized in RF magnetron sputtering comprised In_2_O_3_/Ga_2_O_3_/ZnO in a molar ratio of 1:1:1. The target–substrate distance was fixed at 10 cm. Following IGZO deposition, the films were annealed at 500 °C for 20 min in an ambient atmosphere. The source and drain contacts were fabricated using a double-layer of Ti (10 nm) and Ag (50 nm), with Ag deposition following Ti deposition via e-beam evaporation. Subsequently, the post-annealing of the devices was performed at 100 °C for 100 s in a N_2_ atmosphere. The channel length (L) and width (W) of the IGZO TFTs were 500 and 1000 μm, respectively. A shadow mask was used to designate the channel, source and drain layers. For each experimental condition, five devices were measured, and fifteen devices per batch were processed under identical sputtering and annealing parameters.

### Characterizations

Transmission electron microscopy (TEM), including energy-dispersive X-ray spectroscopy (EDS) mapping and selected area electron diffraction (SAED), was conducted using a TECNAI G2 F30 ST (FEI, USA) to characterize the interface morphology, elemental composition, and thickness of the IGZO thin-film transistors (TFTs). X-ray photoelectron spectroscopy (XPS) was performed using a PHI 5000 VersaProbe system (ULVAC-PHI, Japan) to compare the chemical compositions of on-axis and off-axis IGZO films deposited on Si substrates. The XPS spectra were calibrated using the C 1s peak at 285.0 eV. Based on the XPS results, density functional theory (DFT) simulations were conducted to gain further insights into the electronic structure of IGZO. Electrical measurements of the IGZO TFTs were carried out using a Keithley 4200 semiconductor parameter analyzer. Across multiple fabrication batches, the IGZO films exhibited consistent composition and electrical characteristics, with less than 10% variation in key electrical parameters, confirming uniform performance and reproducible film quality. All electrical and optical measurements were carried out under controlled ambient conditions at 25 ± 5 °C and 45 ± 10% relative humidity. Optical excitation was provided by LEDs at 470, 530, 625, and 850 nm with power densities between 100 and 500 µW cm^-2^. Reference data for coffee sweetness were obtained using the PAL-COFFEE (BX/TDS) refractometer (Atago Co., Ltd., Japan). Also, the coffee Brix measurements were carried out under controlled ambient conditions at 25 ± 5 °C and 45 ± 10% relative humidity. To ensure the accuracy of photocurrent measurements according to sugar concentration and to avoid artifacts caused by thermal variations, all brewed coffee samples were cooled down to room temperature and allowed to reach full thermal equilibrium (25 °C) prior to testing. This protocol ensured that no temperature changes occurred during the measurement, keeping temperature-dependent parameters, such as the refractive index of the samples and the carrier mobility of the a-IGZO channel, constant throughout the evaluation. While broader variations in ambient temperature and humidity may potentially impact the photoresponse, a systematic investigation of these environmental factors is beyond the scope of this study and remains a subject for future investigation, allowing this work to focus strictly on the correlation between NIR photoresponse and sugar concentration. Near-infrared (NIR) spectroscopy was employed to evaluate the sugar content of coffee samples, which were held in place using a custom-designed holder fabricated via 3D printing.

### Calculation method

To predict the generation and role of oxygen related defects in the IGZO(In_2_O_3_:Ga_2_O_3_:ZnO = 1:2:1) system, we performed density functional theory (DFT) calculations with the Vienna ab-initio simulation package (VASP) and the PBEsol functional^39,40^. First, we generated three amorphous models by a melt-quench process with an ab-initio molecular dynamics (MD) simulation. During the MD simulation, an NPT ensemble with a Langevin thermostat, gamma K-point, and 500 eV cut-off energy is used^41^. A reference model includes 15 indium (In), 30 gallium (Ga), 15 zinc (Zn), and 80 oxygen (O) atoms, corresponding to a metal-oxide stoichiometry of In_2_O_3_:Ga_2_O_3_:ZnO = 1:2:1. To study the effects of oxygen non-stoichiometry, additional models are constructed with 78 (oxygen-deficient) or 82 (oxygen-rich) O atoms, while keeping the cation composition constant. In the first stage, both systems are simulated to melt at 3000 K for 12 ps with a time step of 3 fs. After melting, molecular dynamics (MD) simulations are performed to cool the systems from 2000 K to 300 K at a rate of 50 K/ps over 54 ps, using the same 3 fs time step. During the MD simulations, the Γ-point is used for Brillouin zone sampling, and a plane-wave cut-off energy of 500 eV is applied. Following the MD process, geometric optimizations are carried out until the force convergence criterion of 0.01 eV/Å is satisfied. For the structural optimization, a 2 × 2 × 2 Monkhorst-Pack k-point grid, a 500 eV cut-off energy, and the PBEsol exchange–correlation functional are used. Based on the optimized structures, the electronic properties of IGZO with oxygen-related defects were calculated using the HSE06 hybrid functional, a 2 × 2 × 2 k-point mesh, and a cutoff energy of 500 eV. The formation energy (E_Formation_) of oxygen-deficient or oxygen-rich IGZO is evaluated under the assumption that excess or missing oxygen atoms form molecular oxygen (O_2_).

$${\mathrm{E}}_{\mathrm{Formation}} = {\mathrm{E}}(In_{15}Ga_{30}Zn_{15}O_{80}-{\mathrm{n}}) - {\mathrm{E(}}In_{15}Ga_{30}Zn_{15}O_{80}) + n/2\times E(O_{2}).$$

To simulate the charging effect on IGZO, calculations with − 2, − 1, 0, 1, 2 charging states were performed with the same conditions. Formation energies (E_charging_) of charging states are calculated from the following equation^42^.

$$E_{charging} = E(q)-E(n)+q(\mu_{e}+\Delta V)$$

where E(q) is the total energy of the supercell with charge q, E(n) is the total energy of a neutral supercell, $\:{\upmu\:}$_e_ is the electron chemical potential, and $\:{\Delta\:}$V is the energy level shift of the valence band maximum.

## Supplementary Information

Below is the link to the electronic supplementary material.

Supplementary Material 1

## Data Availability

The datasets used and/or analysed during the current study are available from the corresponding author on reasonable request.
