# Supplementary material for: Sputtering-driven formation of interstitial oxygen for intrinsic NIR detection in IGZO phototransistor
Source: Sci Rep. 2026 Feb 25;16:11065. doi: 10.1038/s41598-026-40769-z (PMC13043663; doi:10.1038/s41598-026-40769-z)
Supplement: Supplementary file 1 — Supplementary Material 1 [file 41598_2026_40769_MOESM1_ESM.docx]

Supporting Information

Sputtering-driven formation of interstitial oxygen for intrinsic NIR detection in IGZO phototransistor

*Jinsik Choe**^1,2^, Hyeonmin Bong^1,3^, Huiyeong Lee^1,3^, Dong-Hun Yeo^1^, Sahn Nahm^2^, In Soo Kim^4^, Mann-Ho Cho^3,5^, Kwangsik Jeong^6,*^, Sungjin Park^1,*^*

^1^ Semiconductor Solution Center, Korea Institute of Ceramic Engineering and Technology (KICET), Icheon Branch, Icheon 17303, Republic of Korea
E-mail: [sj1107.park@kicet.re.kr](mailto:sj1107.park@kicet.re.kr)

^2^ Department of Materials Science and Engineering, Korea University, Seoul 02841, Republic of Korea

^3^ Department of Physics, Yonsei University, Seoul, Republic of Korea

^4^ Center for Nanoscale Materials, Argonne National Laboratory, Lemont 60439, United States

^5^ Department of System Semiconductor Engineering, Yonsei University, Seoul 03722, Republic of Korea

^6^ Division of AI Semiconductor, Yonsei University, Wonju, 26493, Republic of Korea
E-mail: [jks0701@yonsei.ac.kr](mailto:jks0701@yonsei.ac.kr)


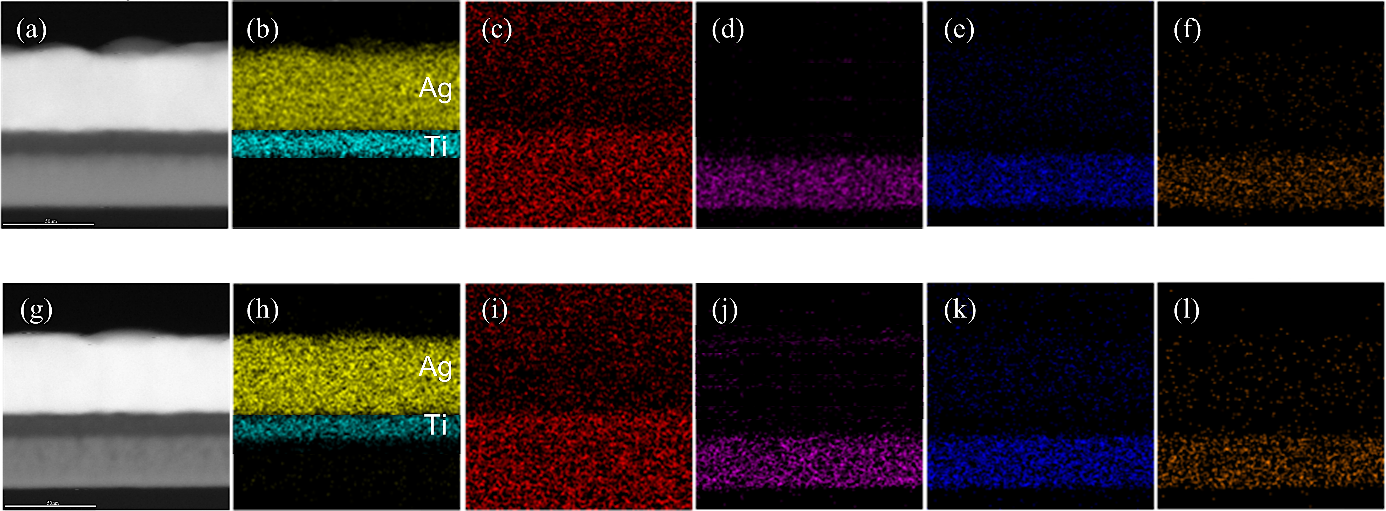


**Figure S1.** Cross-sectional transmission electron microscopy (TEM) and corresponding energy-dispersive X-ray spectroscopy (EDS) elemental mapping images of (a–f) on-axis and (g–l) off-axis IGZO thin-film transistor (TFT) structures. Panels (a) and (g) show low-magnification TEM images of the full device stack, clearly resolving the layered structure including the Ti/Ag top electrodes and the underlying IGZO channel. Elemental distributions of Ag and Ti are shown in (b) and (h), confirming the integrity and uniformity of the metal electrode layers. Panels (c) and (i) show oxygen distribution, while (d)/(j), (e)/(k), and (f)/(l) respectively display the spatial distributions of indium (In), gallium (Ga), and zinc (Zn). All three cationic elements are uniformly distributed within the IGZO layer in both deposition geometries.

**
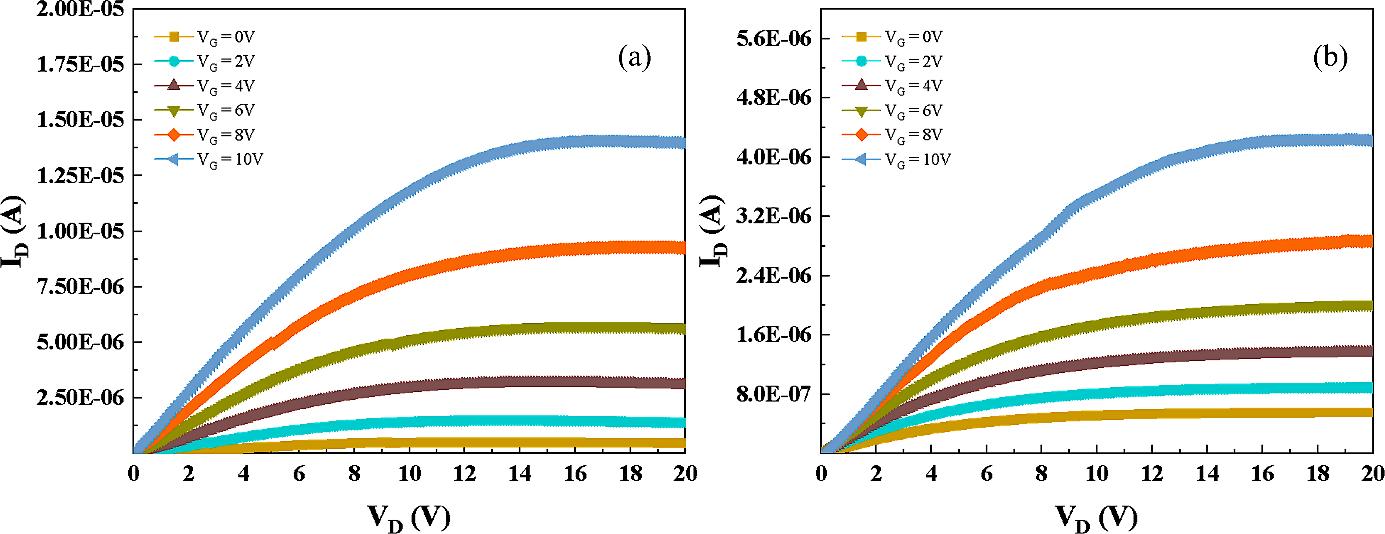
**

**Figure S2.** Output characteristics of IGZO thin-film transistors (TFTs) fabricated by (a) on-axis and (b) off-axis sputtering methods. Drain current (I_d_) was measured as a function of drain voltage (V_d_) at gate voltages (V_g_) ranging from 0 to 10 V in 2 V steps. Both devices exhibit typical n-type transistor behavior with increasing current at higher V_g_ and V_d_. The on-axis device (a) demonstrates a higher I_d_ at each V_g_ compared to the off-axis counterpart (b), indicating improved channel conductivity. This difference is attributed to variations in film stoichiometry and defect states induced by the sputtering geometry, which are further discussed in the main text and correlated with structural and spectroscopic analyses.


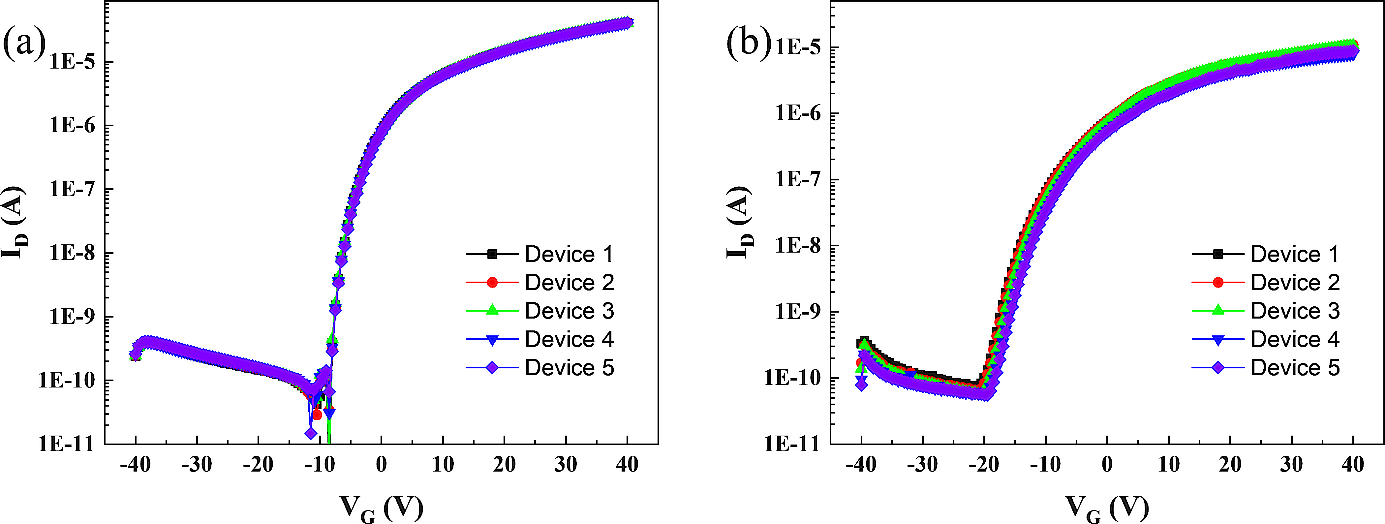


**Figure S3.** Device-to-device reproducibility of transfer characteristics in (a) on-axis and (b) off-axis IGZO phototransistors. Transfer curves (I_d_–V_g_) measured from five independently fabricated devices under identical conditions demonstrate consistent electrical behavior, confirming high reproducibility and uniform film quality.


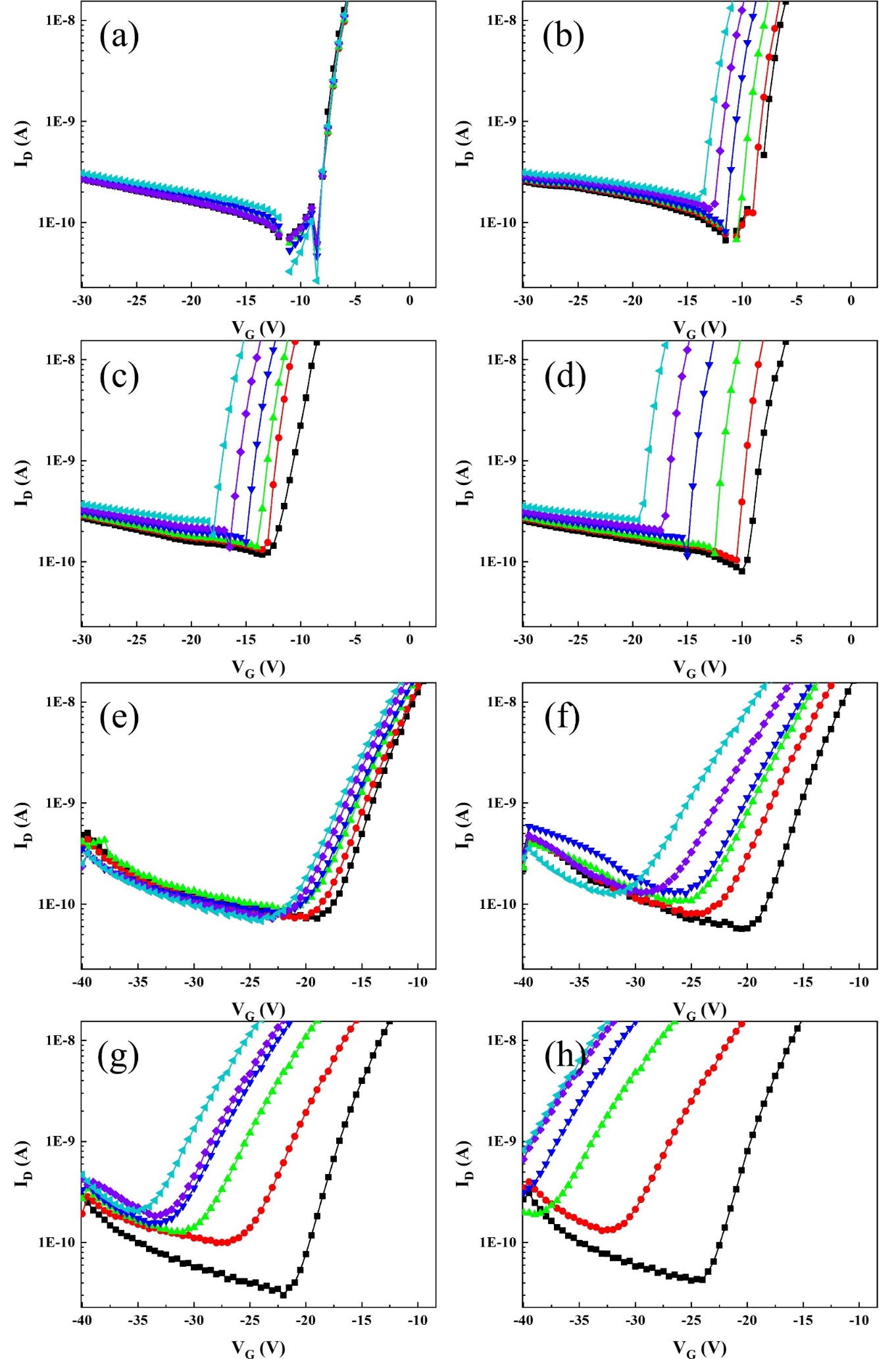


**Figure S4.** Magnified transfer characteristics near the threshold voltage (V_th_) region for on-axis (a–d) and off-axis (e–h) IGZO phototransistors. These plots clearly illustrate the negative shift of V_th_ under (a, e) 850 nm, (b, f) 625 nm, (c, g) 530 nm, and (d, h) 470 nm illumination as a function of incident power density. The increased legibility of these magnified views supports the analysis of the trap-mediated photogating mechanism.


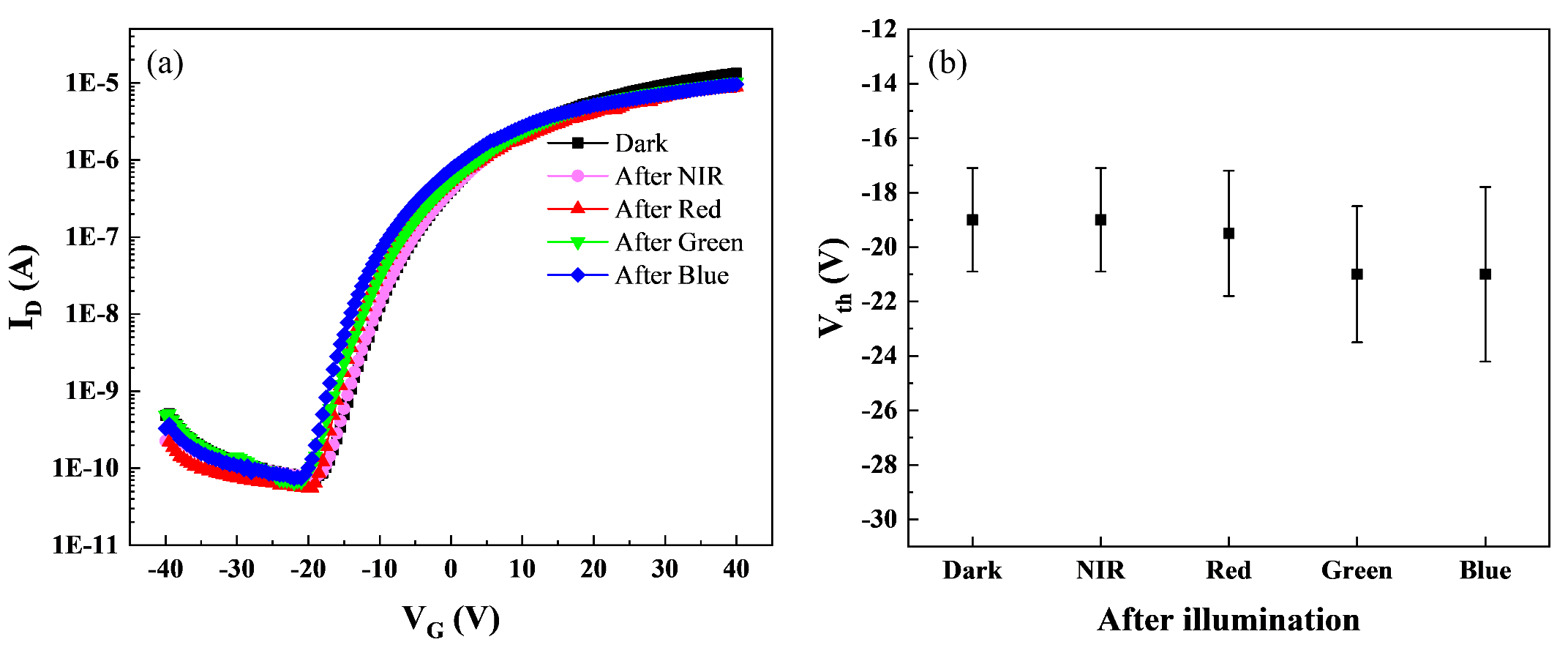


**Figure S5.** Reversibility and dark current recovery of the off-axis IGZO phototransistor after illumination.

(a) Transfer characteristics (V_g_-I_d_) of the off-axis IGZO device measured after NIR (850 nm), red (625 nm), green (530 nm), and blue (470 nm) illumination to verify the recovery of the initial dark state. (b) Statistical analysis showing the average dark current and the corresponding error bars for each wavelength. The data confirms that the dark current remains unchanged after NIR illumination and that any variations following visible light exposure are within the experimental margin of error, demonstrating the excellent reversibility of the device.


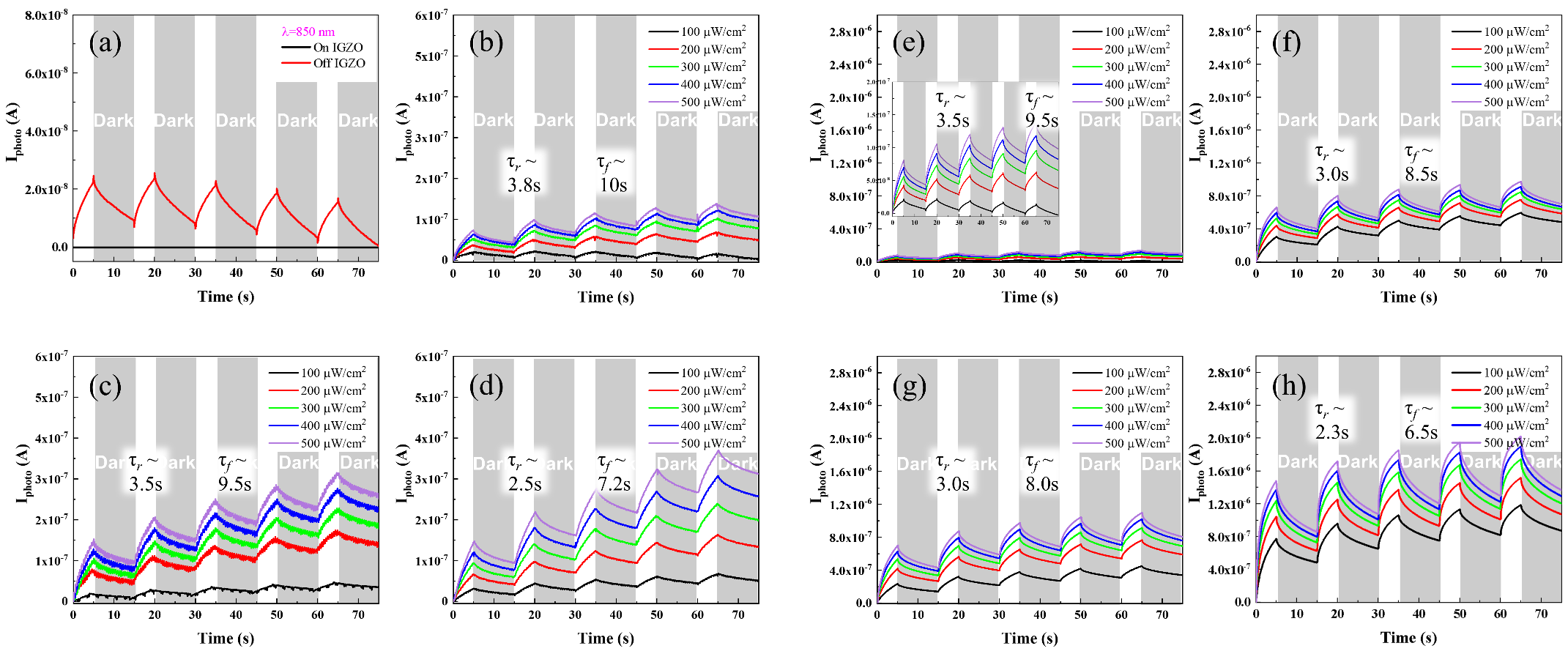


**Figure S6.** Transient photoresponse characteristics of on-axis and off-axis IGZO phototransistors under illumination with different wavelengths and light intensities. (a) Comparison of on-axis and off-axis IGZO devices under 850 nm near-infrared (NIR) illumination at an incident power density of 100 μW/cm². The off-axis device shows a significantly stronger and more stable photocurrent response compared to the on-axis device. (b–d) Transient photocurrent responses of the on-axis IGZO phototransistor under increasing light intensities (100–500 μW/cm²) at visible wavelengths of (b) 625 nm (red), (c) 530 nm (green), and (d) 470 nm (blue), respectively. (e–h) Corresponding photoresponses of the off-axis IGZO phototransistor under the same power densities and wavelengths: (e) 850 nm (NIR), (f) 625 nm (red), (g) 530 nm (green), and (h) 470 nm (blue). All measurements were performed at a gate voltage (V_g_) of 20 V and a drain voltage (V_d_) of 5 V. The light was modulated in 5 s on / 10 s off cycles, repeated for five cycles to evaluate response stability and reproducibility. The enhanced response of the off-axis device, particularly under NIR illumination, is consistent with the defect-state analysis discussed in the main text.


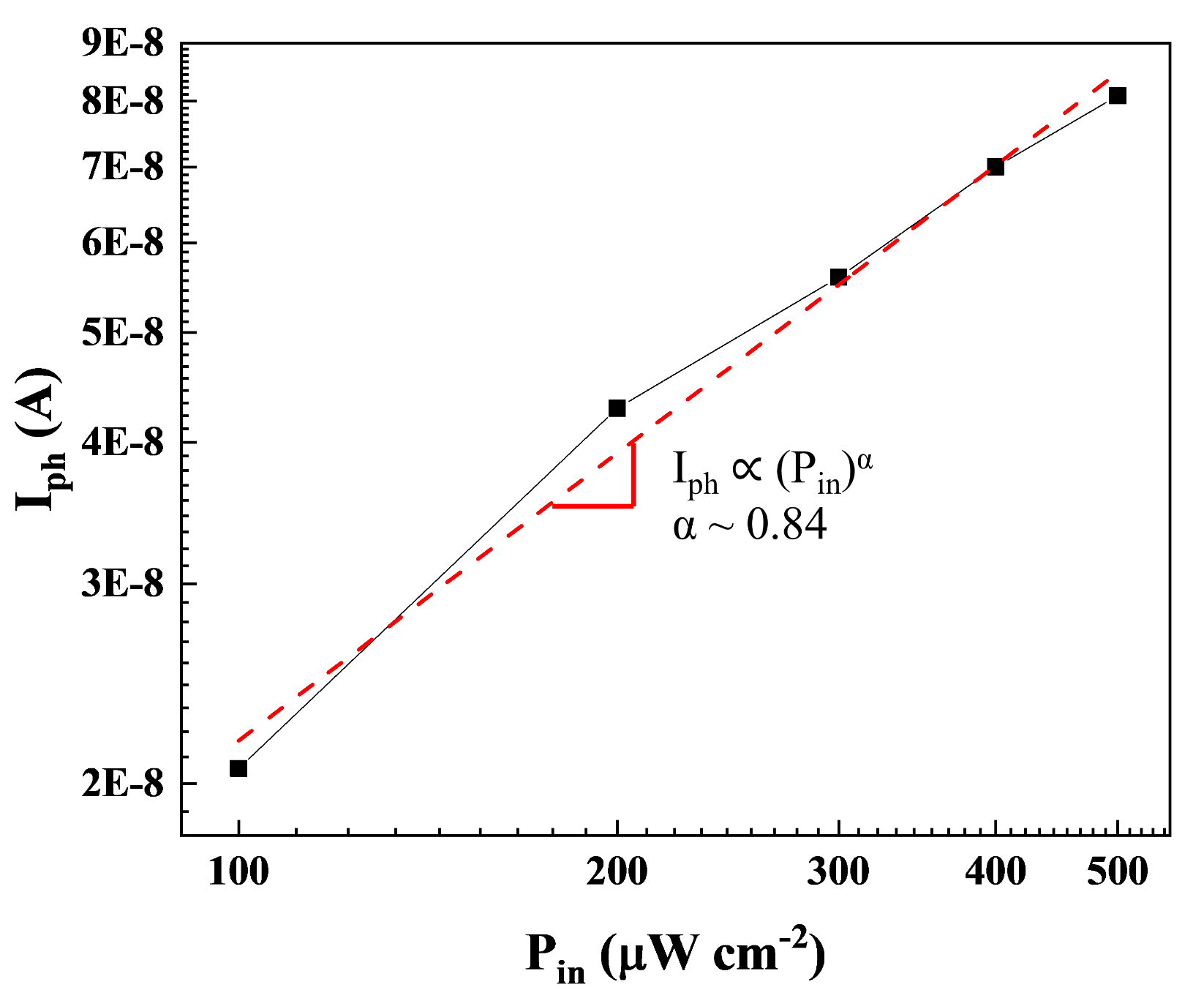


**Figure S7.** Power-dependent photoresponse of the off-axis IGZO phototransistor under NIR illumination.
The photocurrent (I_ph_) exhibits a sublinear dependence on the incident optical power density (P_in_), following the relation *I_ph_ ∝ P_in_^α^* with an extracted exponent of α = 0.84, as obtained from the log–log plot. This behavior indicates the involvement of trap-assisted and photogating-related processes rather than purely band-to-band photogeneration.


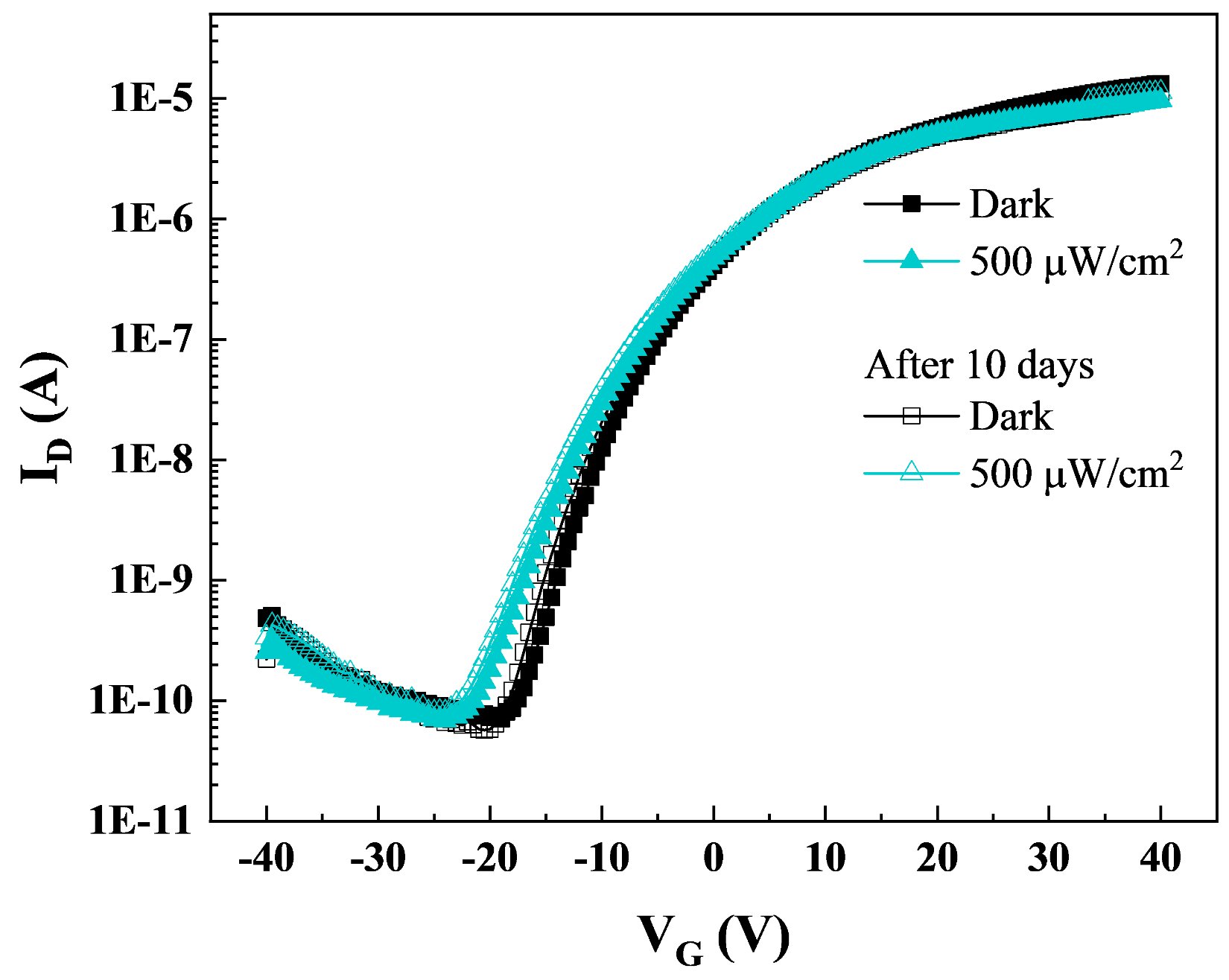


**Figure S8.** Long-term operational stability of the off-axis IGZO phototransistor after 10 days of aging. Transfer characteristics (V_g_-I_d_) of the off-axis IGZO phototransistor measured initially and after 10 days of storage in an ambient atmosphere. The dark state baseline remains remarkably stable, and the NIR-induced negative V_th_ shift is perfectly preserved, confirming that the O_i_-induced states do not undergo relaxation.


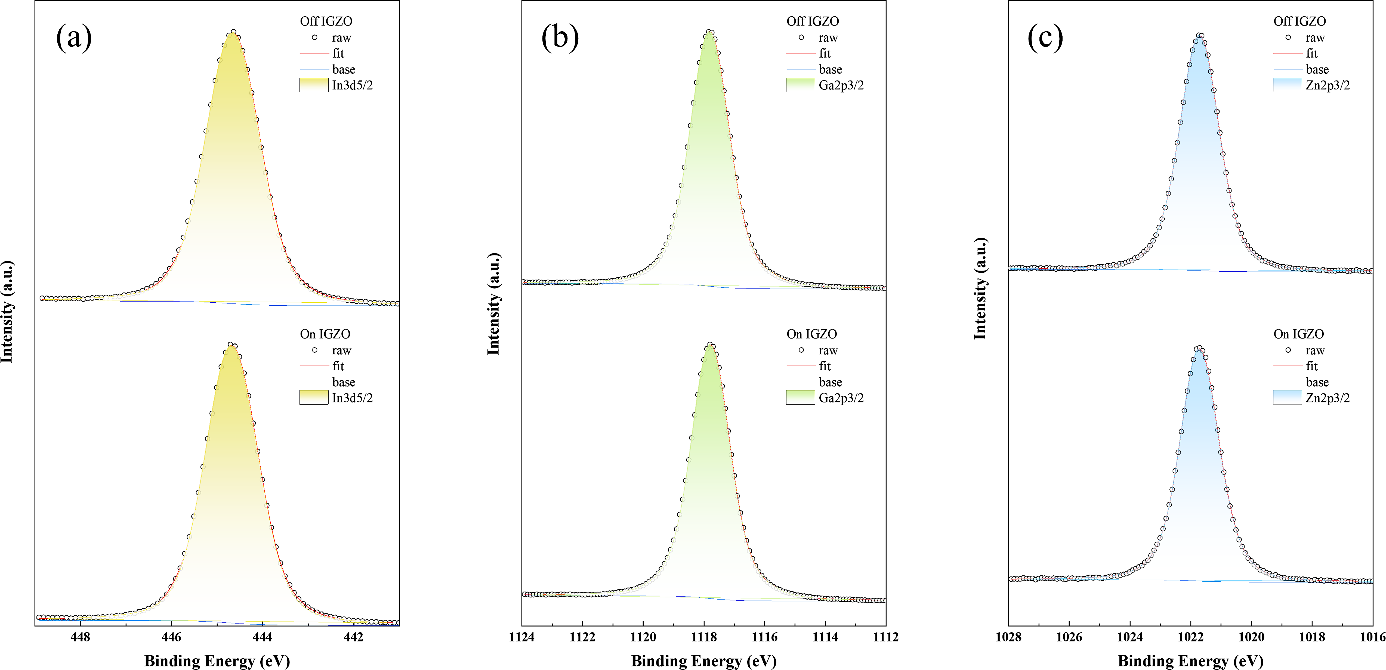


**Figure S9.** High-resolution X-ray photoelectron spectroscopy (XPS) spectra of (a) In 3d_5/2_, (b) Ga 2p_3/2_, and (c) Zn 2p_3/2_ core levels for IGZO films deposited under on-axis (bottom) and off-axis (top) sputtering conditions. The nearly identical peak positions and shapes indicate negligible differences in the chemical states of the constituent elements.


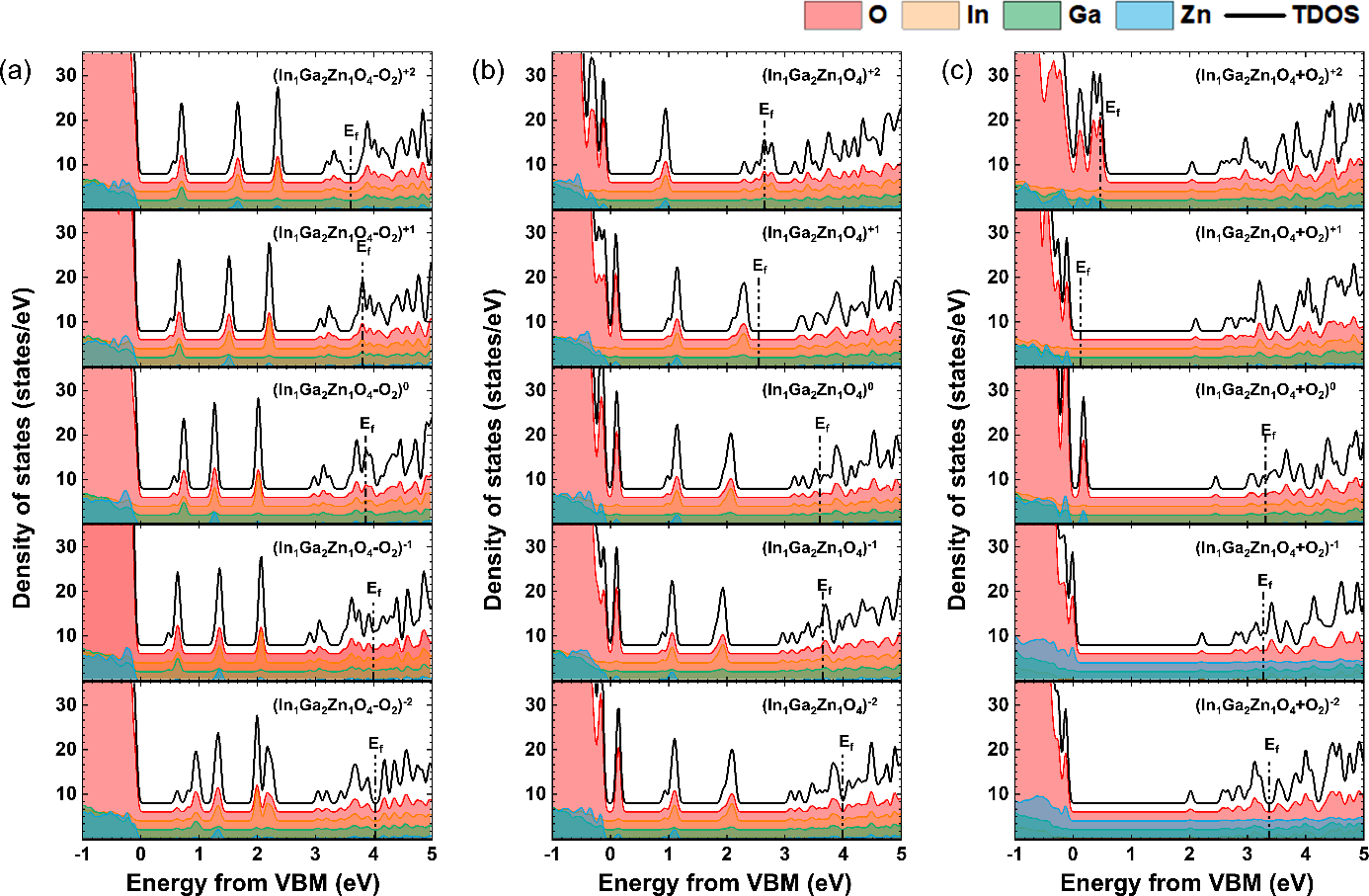


**Figure S10.** Calculated partial and total density of states of (a) In₁Ga₂Zn₁O₄-O₂ (oxygen-deficient), (b) In₁Ga₂Zn₁O₄ (stoichiometric) and (c) In₁Ga₂Zn₁O₄+O₂ (interstitial oxygen) IGZO models at charge states q = +2, +1, 0, –1 and –2 (top → bottom). Energies are plotted relative to the VBM. Oxygen-deficient models instead display deeper mid-gap states, while the stoichiometric phase maintains the widest bandgap. The oxygen-rich configurations form continuous defects state ≤0.5 eV above the VBM in the positive charge state, which acts as a shallow donor-type trap. These shallow defect states can be easily thermally or optically excited, enabling enhanced absorption and photocurrent generation under near-infrared (NIR) illumination. This result supports the observed NIR-responsive behavior in off-axis IGZO phototransistors and is consistent with the photoresponse data presented in the main text. The color legend (upper right) identifies elemental contributions; the solid black curve denotes the total DOS.


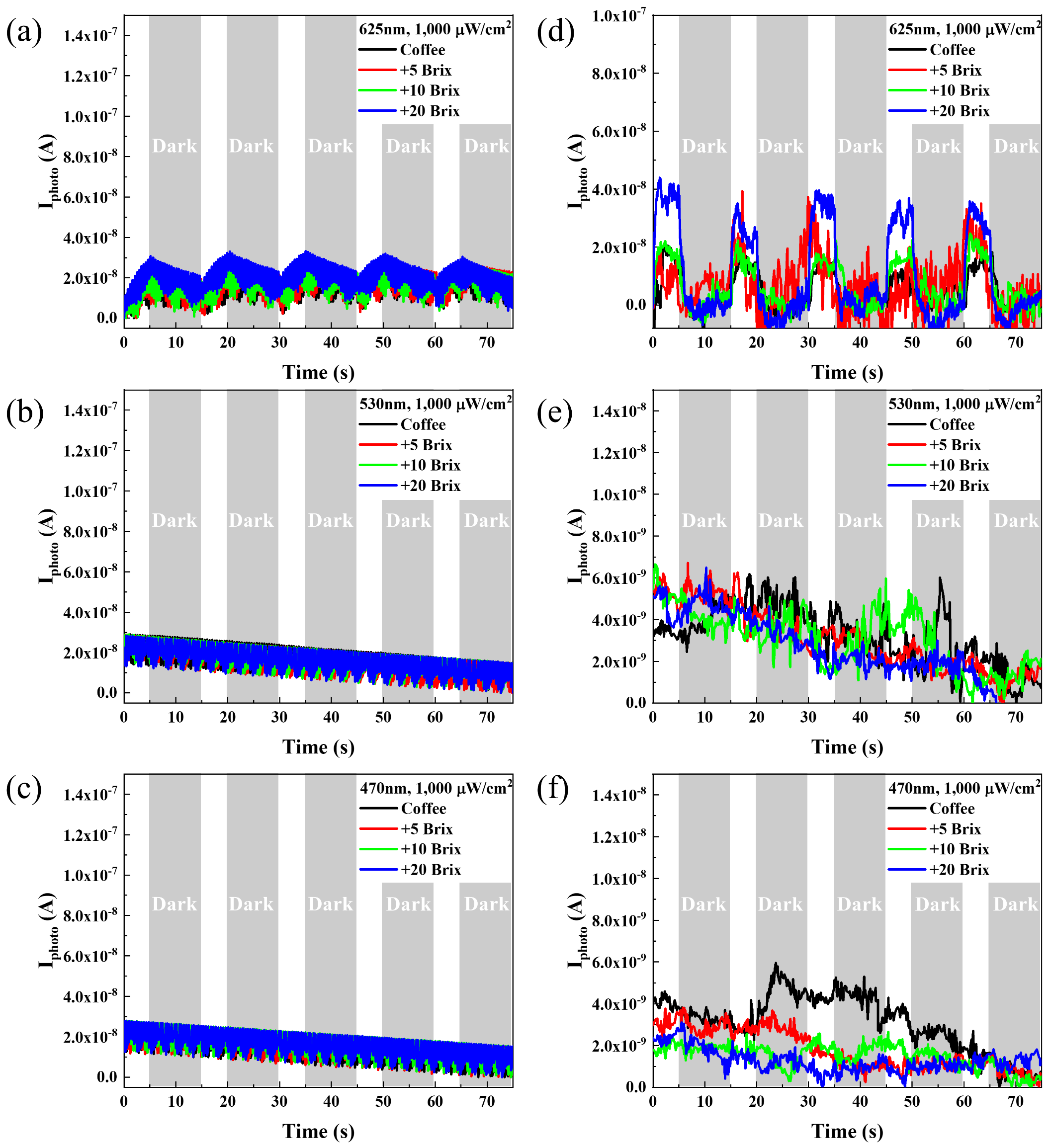


**Figure S11.** Transient photoresponse of IGZO phototransistors to aqueous solutions with varying sugar concentrations (5, 10, and 20 Brix) and brewed coffee, under visible light illumination at red (625 nm), green (530 nm), and blue (470 nm) wavelengths (incident power density = 1000 μW/cm²). (a–c) Photoresponse of the on-axis IGZO device under (a) 625 nm, (b) 530 nm, and (c) 470 nm illumination. While the device shows clear on/off modulation under red light (625 nm), the photocurrent does not vary systematically with Brix concentration, making quantitative discrimination difficult. Under green and blue light, no discernible photocurrent change is observed across different solutions. In the case of near-infrared (NIR, 850 nm) illumination, it was confirmed that there was no response in the on-axis IGZO device, as in the previous experimental results (Figure 2a, S3a). (d–f) Corresponding measurements for the off-axis IGZO device under the same conditions. These results demonstrate that visible-light-based sugar detection using IGZO phototransistors is not viable under the tested conditions.

**Table S1.** Extracted Representative Electrical Parameters of on- and off-axis IGZO device.

|  | V_th_ (V) | μ_sat_ (cm^2^/V·s) | S.S. (V/dec) | I_on_/I_off_ |
| --- | --- | --- | --- | --- |
| On-axis IGZO | -8.5 | 5.4 ± 0.3 | 0.78 ± 0.02 | (1.16 ± 0.05) × 10^5^ |
| Off-axis IGZO | -20 ± 1.0 | 0.6 ± 0.1 | 2.97 ± 0.07 | (1.85 ± 0.15) × 10^5^ |

**Table S2.** Atomic composition (at. %) of on-axis and off-axis IGZO thin films measured by X-ray photoelectron spectroscopy (XPS). The on-axis IGZO sample exhibits a higher relative content of indium and lower oxygen concentration compared to the off-axis counterpart, which shows increased oxygen content indicative of a more oxygen-rich environment. These differences are consistent with the spectroscopic and electrical characteristics discussed in the main text.

|  | In% | Ga% | Zn% | O% |
| --- | --- | --- | --- | --- |
| On-axis IGZO | 13.33 | 24.24 | 8.90 | 53.53 |
| Off-axis IGZO | 11.66 | 24.47 | 9.27 | 54.60 |
